# A longitudinal study on the bacterial quality of baby spinach cultivated in Arizona and California

**DOI:** 10.1128/aem.00553-24

**Published:** 2024-07-12

**Authors:** Sriya Sunil, Tamara Walsky, Mikayla Henry, Leonie Kemmerling, Magdalena Pajor, Xiaodong Guo, Sarah I. Murphy, Renata Ivanek, Martin Wiedmann

**Affiliations:** 1 Department of Food Science, Cornell University, Ithaca, New York, USA; 2 Department of Population Medicine and Diagnostic Sciences, Cornell University, Ithaca, New York, USA; The Pennsylvania State University, University Park, Pennsylvania, USA

**Keywords:** baby spinach, leafy vegetables, produce, bacterial growth, quality, shelf life

## Abstract

In the U.S., baby spinach is mostly produced in Arizona (AZ) and California (CA). Characterizing the impact of growing region on the bacterial quality of baby spinach can inform quality management practices in industry. Between December 2021 and December 2022, baby spinach was sampled after harvest and packaging for microbiological testing, including shelf-life testing of packaged samples that were stored at 4°C. Samples were tested to (i) determine bacterial concentration, and (ii) obtain and identify bacterial isolates. Packaged samples from the Salinas, CA, area (*n* = 13), compared to those from the Yuma, AZ, area (*n* = 9), had a significantly higher bacterial concentration, on average, by 0.78 log_10_ CFU/g (*P* < 0.01, based on aerobic, mesophilic plate count data) or 0.67 log_10_ CFU/g (*P* < 0.01, based on psychrotolerant plate count data); the bacterial concentrations of harvest samples from the Yuma and Salinas areas were not significantly different. Our data also support that an increase in preharvest temperature is significantly associated with an increase in the bacterial concentration on harvested and packaged spinach. A Fisher’s exact test and linear discriminant analysis (effect size), respectively, demonstrated that (i) the genera of 2,186 bacterial isolates were associated (*P* < 0.01) with growing region and (ii) *Pseudomonas* spp. and *Exiguobacterium* spp. were enriched in spinach from the Yuma and Salinas areas, respectively. Our findings provide preliminary evidence that growing region and preharvest temperature may impact the bacterial quality of spinach and thus could inform more targeted strategies to manage produce quality.

**IMPORTANCE:**

In the U.S., most spinach is produced in Arizona (AZ) and California (CA) seasonally; typically, spinach is cultivated in the Yuma, AZ, area during the winter and in the Salinas, CA, area during the summer. As the bacterial quality of baby spinach can influence consumer acceptance of the product, it is important to assess whether the bacterial quality of baby spinach can vary between spinach-growing regions. The findings of this study provide insights that could be used to support region-specific quality management strategies for baby spinach. Our results also highlight the value of further evaluating the impact of growing region and preharvest temperature on the bacterial quality of different produce commodities.

## INTRODUCTION

Most fresh spinach available for consumption in the U.S. is cultivated domestically. In 2021, the U.S. produced approximately 693 million pounds of spinach (1) while importing approximately 52 million pounds (2). As spinach is a cool season crop with optimal germination and growth within 12°C–25°C (3, 4), it is grown in various regions across the U.S. to accommodate seasonal changes in temperature. Specifically, spinach is cultivated seasonally in Arizona (AZ) and California (CA), with these two states accounting for over 96% of the spinach produced in the U.S. in 2021 (2). In AZ, spinach is typically cultivated in Yuma during winter (e.g., November–March) (5). In CA, the region of cultivation varies seasonally (6), with most production occurring in (i) the central coast of CA (e.g., Monterey County, which includes the Salinas Valley) during summer (e.g., April–October) and (ii) the desert region of CA (e.g., the Imperial Valley) during winter. Thus, spinach production typically occurs in AZ and the nearby desert regions of CA during winter, and in the central coast and other regions of CA during summer.

As spinach is mainly grown in two areas of the U.S., there is a need to understand whether growing region impacts the bacterial quality of baby spinach. Many factors can influence the microbiological characteristics (e.g., bacterial concentration) of leafy vegetables such as baby spinach, including (i) cultivar (7), (ii) preharvest environmental conditions (8, 9), and (iii) agricultural and postharvest handling practices (10–13). These factors can subsequently impact the development of sensory defects, which limit consumer acceptance of the product (14, 15). While management practices can control for the effect of some factors (e.g., choice of cultivar) on the postharvest quality of baby spinach, they can only limit the effect of other factors including those that may be associated with the growing region (e.g., weather). Thus, characterizing the impact of growing region on the bacterial quality of produce will provide valuable information for developing effective postharvest quality management strategies.

Prior research has demonstrated that the growing region can impact the bacterial quality of fresh produce. A study (16), conducted in Korea, demonstrated that the growing region impacts the microbiota of broccoli and that the region-based difference in the broccoli microbiota could be attributed to differences in weather. Specific to the U.S., a prior study (17) provided evidence that lettuce sampled in the summer (i.e., cultivated in Monterey County, CA), compared to that sampled in the winter (i.e., cultivated in Yuma, AZ, and Imperial County, CA), had (i) a higher concentration of culturable bacteria and (ii) different bacterial populations (determined using a culture-independent approach); however, as this study tested harvested lettuce, it is unclear whether this region-based difference in bacterial quality can persist after packaging, at which point it could differentially affect consumer acceptance. Thus, the aim of this study was to compare the bacterial quality of harvested and packaged baby spinach across key growing regions. The findings of this study will provide insight into whether industry should consider region-specific quality management strategies for commodities such as baby spinach.

## RESULTS

### Sample collection

Across all 27 samplings conducted during this study (Table 1), we collected 23 harvest samples, including (i) 8 from Yuma, AZ and 1 from Imperial Valley, CA (these samples will be referred to as “Yuma, AZ, area” samples as these regions are in close proximity and as spinach is cultivated at the same time in these regions), (ii) 13 from Salinas Valley, Monterey County, CA (these will be referred to as “Salinas, CA, area” samples), and (iii) 1 from Florida (FL). We also collected 25 packaged samples, including (i) 9 from the Yuma, AZ, area (8 from Yuma, AZ and 1 from Imperial Valley, CA), (ii) 13 from the Salinas, CA, area, and (iii) 1 each from FL and Georgia (GA); we were unable to determine the growing region for one packaged sample (0922_B), which was thus excluded from statistical analysis. Fewer harvest samples were collected, compared to packaged samples, due to logistical constraints. For 17 of the 27 rounds of sampling, harvest and packaged samples were collected from the same order (i.e., lot); for the 10 remaining rounds of sampling, we either collected (i) harvest and packaged samples from different orders during the same month (collected within 14 days of each other) or (ii) only harvest or packaged samples. In addition to excluding data from the 0922_B packaged sample from statistical analysis, we also excluded data from (i) the 0722_B harvest sample due to a delay in sample shipment to Ithaca, NY and (ii) the aerobic plate count (APC) and total gram-negative count (GN) tests for the 1222_A harvest sample due to a lab error.

**TABLE 1:** Summary of the samples collected during this study

		Sample^*f*^		
Time of sampling, YYYY_MM (sampling ID)^*a*^	State of origin (growing region)	Harvest	Packaged	Harvest and packaged sample from the same order^*f*^
2021_12 (1221_A)	FL (NA)^*b*^	Y	Y	Y
2022_01 (0122_A)	AZ (Yuma, AZ, area)	N	Y	NA
2022_01 (0122_B)	AZ (Yuma, AZ, area)	Y	Y	Y
2022_02 (0222_A)	AZ (Yuma, AZ, area)	Y	Y	Y
2022_02 (0222_B)	AZ (Yuma, AZ, area	Y	Y	Y
2022_03 (0322_A)	AZ (Yuma, AZ, area)	Y	Y	Y
2022_03 (0322_B)	AZ (Yuma, AZ, area)	Y	Y	Y
2022_04 (0422_A)^ab^	AZ (Yuma, AZ, area)	Y	Y	N
2022_04 (0422_B)^ab^	CA (Salinas, CA, area)	Y	Y	Y
2022_05 (0522_A)^ab^	CA (Salinas, CA, area)	Y	Y	Y
2022_05 (0522_B)^a^	CA (Salinas, CA, area)	Y	Y	Y
2022_05 (0522_C)^a^	CA (Salinas, CA, area)	Y	Y	Y
2022_06 (0622_A)^a^	CA (Salinas, CA, area)	Y	Y	Y
2022_06 (0622_B)	CA (Salinas, CA, area)	Y	Y	N
2022_07 (0722_A)	CA (Salinas, CA, area)	Y	Y	N
2022_07 (0722_B)^*c*^	CA (Salinas, CA, area)	Y	N	NA
2022_08 (0822_A)	CA (Salinas, CA, area)	Y	Y	Y
2022_08 (0822_B)	CA (Salinas, CA, area)	N	Y	NA
2022_09 (0922_A)	CA (Salinas, CA, area)	Y	Y	Y
2022_09 (0922_B)	CA (Salinas, CA, area)	Y	Y^*d*^	N
2022_10 (1022_A)	CA (Salinas, CA, area)	Y	Y	Y
2022_10 (1022_B)	CA (Salinas CA, area)	Y	Y	Y
2022_11 (1122_A)	CA (Salinas, CA, area)	N	Y	NA
2022_11 (1122_B)	GA (NA)^*b*^	N	Y	NA
2022_12 (1222_A)^*e*^	CA (Yuma, AZ, area)	Y	Y	Y
2022_12 (1222_B)	AZ (Yuma, AZ, area)	Y	Y	Y
2022_12 (1222_C)	AZ (Yuma, AZ, area)	Y	N	NA

^*a*^ Sampling IDs with the superscript “a” indicate packaged samples that were tested on days initial, 7, 14, 21, and 28; P samples from all remaining sampling IDs were tested on days initial, 7, 12, 17, and 22. Sampling IDs with the superscript “b” indicate P samples that were not tested for the entire duration of shelf life due to an incubator malfunction. These P samples were tested up until the following days of shelf life: (i) day 21 for 0422_A, (ii) day 14 for 0422_B, and (iii) day 7 for 0522_A.

^*b*^ NA, not applicable.

^*c*^ The H samples from the 0722_B order were excluded from analysis due to a delay in transit (i.e., 4 days in transit) from the grower to Ithaca, NY.

^*d*^ We were unable to collect field-level metadata for the P sample (i.e., packaged sample) from sampling 0922_B, so this sample was excluded from analysis due to the lack of field-level metadata (e.g., date of harvest and weather data).

^*e*^ We excluded the data from the APC and GN test for 1222_A, as these tests were incubated at the wrong temperature. Additionally, the 1222_A harvest and packaged samples were obtained from Imperial Valley, CA and were grouped with the samples from Yuma, AZ for analyses.

^*f*^ Y=yes, N=no; “yes” and “no” indicate whether we collected harvest and/or packaged samples during a sampling (e.g., for the 0122_A sampling, we collected a packaged sample, but not a harvest sample). "no" in the "Harvest and packaged sample from the same order" column indicates that the harvest and packaged samples, collected during a given sampling, came from different orders (i.e., lots) (e.g., for the 0422_A sampling, the harvest and packaged samples were collected from different orders). NA represents "not applicable" in the "Harvest and packaged sample from the same order" column; this indicates samplings in which we collected either a harvest sample or a packaged sample.

### The bacterial concentration of harvested baby spinach did not differ by growing region

**Fig 1:**
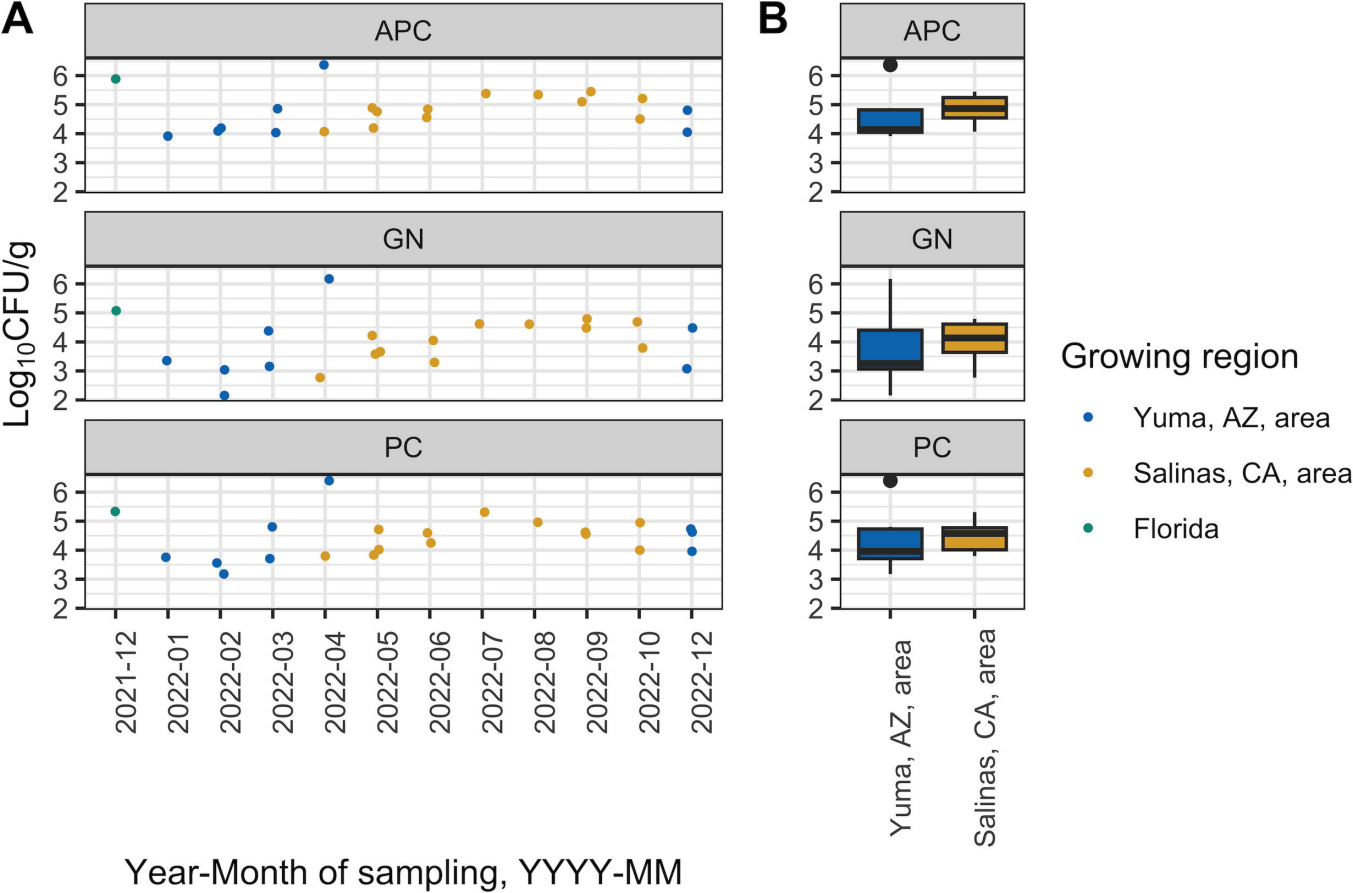
Bacterial concentration of baby spinach (in log_10_ CFU/g; y-axis) sampled at harvest, by test. For plot A (scatterplot), the x-axis is the time of sampling (YYYY-MM), and y-axis is the bacterial concentration of a sample. For plot B (boxplot), the x-axis represents the growing region, and the y-axis is bacterial concentration; this plot only represents samples from the Yuma, AZ, area and the Salinas, CA, area. Color represents growing region. The data are depicted separately by test, as indicated by the following labels: (i) aerobic plate count, (ii) total gram-negative count, and (iii) psychrotolerant count. The following samples were excluded from this plot: (i) the 0722_B harvest sample, which was delayed in shipment to Ithaca, NY, and (ii) the APC and GN test of the 1222_A harvest sample due to a lab error.

The median [interquartile range (IQR)] APC of the combined harvest samples was 4.81 (4.19–5.21) log_10_ CFU/g. Harvest samples from the Yuma, AZ, area and the Salinas, CA, area had a median (IQR) APC of 4.14 (4.05–4.82) and 4.87 (4.54–5.24) log_10_ CFU/g, respectively (Fig. 1). The harvest sample from FL had an APC of 5.89 log_10_ CFU/g. Typically for harvest samples, psychrotolerant count (PC) and GN were lower than APC; for example, the median (IQR) PC and GN of the combined harvest samples were 4.58 (3.87–4.79) and 4.05 (3.29–4.61) log_10_ CFU/g, respectively. A linear mixed effects model showed that the interaction of test (APC, PC, and GN) and growing region (i.e., Yuma and Salinas areas) was not significant, which indicates that we would observe comparable differences in the bacterial concentration of spinach by growing region regardless of which test (i.e., APC or PC) was selected for further analysis. Thus, a linear fixed effects model was fit to the APC of the harvest samples (H model; see Table 2). Following stepwise backward elimination, the following independent variables were retained in the H model: (i) growing region and (ii) time from harvest until testing. Based on the H model, the time from harvest until testing was borderline significant (*P* = 0.10); on average, a 1-h increase in the time between harvest and testing was associated with a 0.01 log_10_ CFU/g increase in the APC of the harvest samples. After controlling for the time from harvest until testing, harvest samples from the Salinas, CA, area, on average, have a 0.44 log_10_ CFU/g higher APC than the Yuma, AZ, area harvest samples; however, this difference was not significant (*P* = 0.13).

**TABLE 2:** Summary of the mixed effects models, fit to data from the harvest and packaged samples

Fixed effect	Estimate	Std. error	df^*a*^	*t* Value	Pr(>|*t*|)	Sig. level^*b*^
H model; APC, without weather						
Intercept	3.85	0.45	NA	8.52	1.53 × 10^−7^	***
Salinas, CA, area^*c*^	0.44	0.28	NA	1.58	0.13	NS
Hours from harvest to testing	0.01	0.01	NA	1.72	0.10	.
H model; APC, with weather						
Intercept	2.01	0.53	NA	3.78	1.63 × 10^−3^	**
Salinas, CA, area^*c*^	0.20	0.20	NA	0.97	0.35	NS
Hours from harvest to testing	0.02	0.01	NA	2.90	0.01	**
Mean temperature ˚C, 0–24 h pth	0.12	0.03	NA	4.33	5.17 × 10^−4^	***
P model; APC, without weather (APC model)						
Intercept	5.72	0.35	18.90	16.22	1.53 × 10^−12^	***
Salinas, CA, area^*c*^	0.78	0.19	18.77	4.03	7.24 × 10^−4^	***
Poly(day, 2)linear^*d*^	8.90	0.31	80.99	29.19	<2 x 10^−16^	***
Poly(day, 2)quadratic^*d*^	−2.94	0.31	81.18	−9.45	1.01 × 10^−14^	***
Days, harvest to arrival	0.31	0.08	19.14	4.03	6.97 × 10^−4^	***
P model; APC, with weather (APC model)						
Intercept	5.64	0.27	18.15	20.84	3.98 × 10^−14^	***
Salinas, CA, area^*c*^	0.56	0.16	18.23	3.53	2.36 × 10^−3^	**
Poly(day, 2)linear^*d*^	8.90	0.30	81.79	29.26	<2 x 10^−16^	***
Poly(day, 2)quadratic^*d*^	−2.93	0.31	82.13	−9.43	9.80 × 10^−15^	***
Days, harvest to arrival	0.20	0.07	18.35	3.10	6.12 × 10^−3^	**
Minimum temperature ˚C, 73–168 h pth	0.08	0.02	18.09	3.83	1.21 × 10^−3^	**
P model; PC, without weather (PC model)						
Intercept	6.26	0.43	19.19	14.71	6.65 × 10^−12^	***
Salinas, CA, area^*c*^	0.67	0.23	19.08	2.87	9.84 × 10^−3^	**
Poly(day, 2)linear^*d*^	9.71	0.33	81.10	29.18	<2 x 10^−16^	***
Poly(day, 2)quadratic^*d*^	−3.61	0.34	81.23	−10.62	<2 x 10^−16^	***
Days, harvest to arrival	0.29	0.09	19.39	3.15	5.22 × 10^−3^	**
P model; PC, with weather (PC model)						
Intercept	6.18	0.38	18.29	16.25	2.57 × 10^−12^	***
Salinas, CA, area^*c*^	0.47	0.22	18.37	2.11	0.05	*
Poly(day, 2)linear^*d*^	9.71	0.33	81.37	29.19	<2 x 10^−16^	***
Poly(day, 2)quadratic^*d*^	−3.58	0.34	81.50	−10.56	<2 x 10^−16^	***
Days, harvest to arrival	0.19	0.09	18.43	2.11	0.05	*
Minimum temperature ˚C, 73–168 h pth	0.07	0.03	18.28	2.45	0.03	*

^*a*^ Degrees of freedom.

^*b*^ The significance level represents the *P*-value: (i) *** for 0 ≤ *P* ≤ 0.001, (ii) ** for 0.001 < *P* ≤ 0.01, (iii) * for 0.01 < *P* ≤ 0.05, and (iv) . for 0.05 < *P* ≤ 0.1. NS represents *P*-values >0.10.

^*c*^ The Salinas, CA, area represents samples from Monterey County, CA. The reference level, for growing region, in these models was “Yuma, AZ, area,” which represents samples from Yuma, AZ and the single sample from the Imperial Valley, CA.

^*d*^ Day was included as a second-degree polynomial using the poly() function in R to achieve orthogonal polynomial contrasts. Poly(day, 2)linear and poly(day, 2)quadratic represent, respectively, the first-order and second-order polynomial of day, which are orthogonal to the zero-order polynomial of day.

### Baby spinach cultivated in the Salinas, CA, area had a higher bacterial concentration over shelf life, as compared to baby spinach from the Yuma, AZ, area

**Fig 2:**
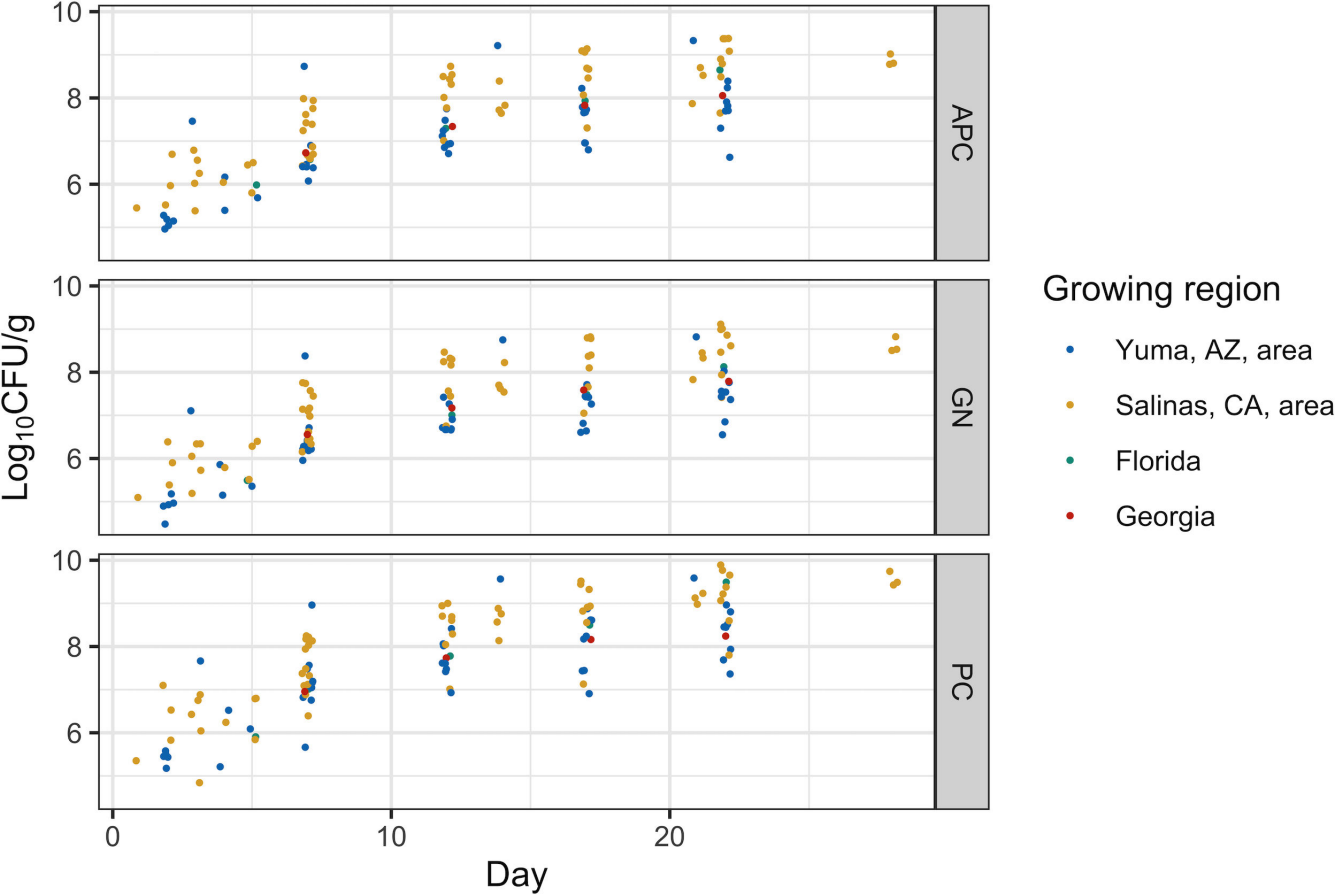
Bacterial concentration of baby spinach sampled after packaging (in log_10_ CFU/g; y-axis), by the day of shelf life and test. The x-axis represents day in shelf life, with day 0 representing the day of packaging. The y-axis represents the bacterial concentration of a sample (i.e., biological replicate), on a given day of shelf life. Color represents growing region. The data are depicted separately by test, as indicated by the following labels: (i) aerobic plate count, (ii) total gram-negative count, and (iii) psychrotolerant count. The 0922_B packaged sample was not included in this plot, as we were unable to collect information on its location of cultivation.

Packaged samples from the Salinas, CA, area had a higher bacterial concentration over shelf life, compared to packaged samples from the Yuma, AZ, area (Fig. 2). For example, on day 7 (D7) of shelf life, packaged samples from the Salinas, CA, area and the Yuma, AZ, area had a median (IQR) APC of 7.24 (6.70–7.62) and 6.46 (6.40–6.61) log_10_ CFU/g, respectively (Fig. S1). For the single packaged sample from each FL and GA, APC on D7 of shelf life was 6.57 and 6.73 log_10_ CFU/g, respectively. The median (IQR) APC of all packaged samples on D7 of shelf life was 6.71 (6.54–7.40) log_10_ CFU/g. For packaged samples, PC was typically higher than APC and GN; for example, the median (IQR) PC and GN of packaged samples, on D7 of shelf life, were 7.26 (7.00–7.96) and 6.51 (6.30–7.15) log_10_ CFU/g, respectively. As a linear mixed effects model showed that the interaction of test (i.e., for APC and PC) and growing region was significant (*P* < 0.05), separate linear mixed effects models were fit to the APC (APC model) and PC (PC model) data of the packaged samples (Table 2). After stepwise backward elimination, which was conducted separately for the APC and PC models, the following independent variables were retained in both models: (i) growing region, (ii) day of shelf life, and (iii) time from harvest until arrival at the processing facility. The time from harvest until arrival at the processing facility had a significant effect (*P* < 0.01) on the APC and PC of the packaged samples; each additional day between harvest and arrival at the processing facility was associated with, on average, a 0.31 and 0.29 log_10_ CFU/g increase in the APC and PC of the packaged samples, respectively (Table 2). After accounting for the day of shelf life and the time from harvest until arrival at the processing facility, packaged samples from the Salinas, CA, area had a bacterial concentration that was significantly higher, on average, than that of packaged samples from the Yuma, AZ, area by (i) 0.78 log_10_ CFU/g (*P* < 0.01), based on the APC model, and (ii) 0.67 log_10_ CFU/g (*P* < 0.01), based on the PC model. A post hoc sample size calculation was conducted, using the data from the packaged samples and the APC and PC models, to identify the minimum number of samples that are necessary to replicate this finding; this analysis indicated that to replicate this study, with an alpha of 0.05 and power of 80%, would require a minimum of (i) 7 packaged samples each from the Salinas, CA, area and the Yuma, AZ, area (to replicate the findings of the APC model), and (ii) 12 packaged samples from Salinas, CA, area and 8 packaged samples from the Yuma, AZ, area (to replicate the findings of the PC model).

To assess whether bacterial concentration at harvest was associated with bacterial concentration over shelf life, we fit P models (i.e., models for packaged samples) to a subset of data (i.e., data from the 17 rounds of sampling, during which harvest and packaged samples were collected from the same order or lot). Based on these “reduced P models,” which were fit separately to APC and PC data, the bacterial concentration of baby spinach at harvest was not significantly (*P* > 0.05) associated with bacterial concentration over shelf life. As the time between harvest and testing had a borderline significant effect on the APC of the harvest samples, we attempted to “normalize” for this effect in the reduced P model by assuming (i) all harvest samples used in the reduced P model had a time from harvest until testing of 36.75 h (i.e., the median time from harvest until testing) and (ii) each additional hour between harvest until testing increased APC by 0.01 log_10_ CFU/g (based on the H model). Even after “normalizing” for the time from harvest until testing, APC at harvest was not significantly associated with APC over shelf life.

We fit primary growth models to the APC and PC of the packaged samples (Fig. S2 and S3), which allowed us to obtain the following parameters to describe bacterial growth on packaged spinach: (i) *N*_0_, which is the initial bacterial concentration (i.e., bacterial concentration on the day of packaging, or day 0 of shelf life), (ii) *μ*_max_, which is the maximum specific growth rate, and (iii) *N*_max_, which is the bacterial concentration at stationary phase. We were unable to accurately fit primary growth models to two packaged samples (0422_B and 0522_A) that could not be tested over shelf life due to an incubator failure; thus, we used the primary growth models fit to the remaining packaged samples (*n* = 22; 9 from the Yuma, AZ, area, 11 from the Salinas, CA, area, and 1 from each FL and GA) for subsequent analysis. As we had shelf-life data for the packaged samples over a fixed time frame (i.e., from the day samples arrived in Ithaca until 22 or 28 days postpackaging), we were unable to directly estimate *N*_0_ from shelf-life data as we did not measure bacterial concentration on the day of packaging. In other words, the *N*_0_ from our primary growth models represents the starting concentration that best explains data observed later in shelf life for our packaged samples while assuming a fixed *μ*_max_ and no lag phase. Similarly, our estimates for *N*_max_ were “extrapolated” for samples of packaged spinach, as there were some instances when the bacteria (i.e., APC or PC) did not reach stationary phase by the end of shelf-life testing. For each APC and PC, the difference in bacterial concentration between the last day and the second-to-last day of shelf-life testing was (i) <0.50 log_10_ CFU/g for 18 packaged samples and (ii) >0.50 log_10_ CFU/g for 4 samples, suggesting that most samples reached *N*_max_ by the end of shelf-life testing (see Fig. S2 and S3). Samples with a difference in bacterial concentration of >0.50 log_10_ CFU/g between the last and second-to-last day of shelf-life testing were (i) based on APC data: 1221_A (FL), 0222_B (Yuma, AZ, area), 0522_B (Salinas, CA, area), and 1222_A (Yuma, AZ, area) and (ii) based on PC data: 1221_A (FL), 0522_B (Salinas, CA, area), 1122_A (Salinas, CA, area), and 1222_A (Yuma, AZ, area).

Overall, the median (IQR) growth parameters, based on primary growth models fit to the APC of the packaged spinach, were (i) *N*_0_ (unit log_10_ CFU/g): 4.81 (4.57–5.00) for the Yuma, AZ, area and 5.56 (5.14–6.01) for the Salinas, CA, area, (ii) *μ*_max_ [unit ln(CFU/g)day^−1^]: 0.55 (0.41–0.69) for the Yuma, AZ, area and 0.51 (0.41–0.64) for the Salinas, CA, area, and (iii) *N*_max_ (unit log_10_ CFU/g): 7.85 (7.74–8.01) for the Yuma, AZ, area and 8.87 (8.73–9.24) for the Salinas, CA, area (see Table 3 for parameters of the growth models for all growing regions). When we used the growth parameters to calculate time to 7 log_10_ CFU/g for packaged samples (Table 3), the median (IQR) time to 7 log_10_ CFU/g, for packaged samples, was (i) 10.2 (9.3–11.0) days for the Yuma, AZ, area and 6.1 (5.0–8.7) days for the Salinas, CA, area, based on APC, and (ii) 7.5 (6.4–7.8) days for the Yuma, AZ, area and 5.8 (3.6–6.1) days for the Salinas, CA, area, based on PC. We were unable to calculate the time to 7 log_10_ CFU/g for two samples (0322_B and 0522_B) that had, respectively, an (i) *N*_max_ that was lower and (ii) *N*_0_ that was higher than 7 log_10_ CFU/g. Mann-Whitney *U* tests were used to compare, by growing region, (i) the parameters of the primary growth models, and (ii) time to 7 log_10_ CFU/g; the Holm method of adjustment was used to account for two comparisons due to the use of two data sets (i.e., APC and PC) for each hypothesis (e.g., the null hypothesis that *N*_0_ did not vary by growing region). Packaged samples from the Salinas, CA, area had a significantly higher *N*_max_ compared to those from the Yuma, AZ, area, based on primary growth models fit to the APC (*P* = 0.01) and PC (*P* = 0.04) data; however, *N*_0_, *μ*_max_, and time to 7 log_10_ CFU/g did not differ significantly by growing region (*P* > 0.05, based on estimates from primary growth models fit to APC and PC data).

**TABLE 3:** The median (interquartile range) of growth parameters and time to 7 log_10_ CFU/g for packaged spinach samples by growing region and microbiological test

		Parameter			
Growing region and microbiological test	Number of samples	*N*_0_, log_10_CFU/g	*µ*_max_, (lnCFU/g)day^−1^	*N*_max_, log_10_ CFU/g	Days to 7 log_10_ CFU/g
Yuma, AZ, area^*a*^	9				
APC		4.81 (4.57–5.00)	0.55 (0.41–0.69)	7.85 (7.74–8.01)	10.2 (9.3–11.0)
PC		4.93 (4.56–5.25)	0.60 (0.54–0.81)	8.34 (7.64–8.64)	7.5 (6.4–7.8)
Salinas, CA, area	11^*b*^				
APC		5.56 (5.14–6.01)	0.51 (0.41–0.64)	8.87 (8.73–9.24)	6.1 (5.0–8.7)
PC		5.39 (5.17–6.15)	0.64 (0.52–0.78)	9.24 (8.96–9.54)	5.8 (3.6–6.1)
Florida	1				
APC		5.31	0.37	9.03	10.5
PC		5.23	0.47	9.87	8.7
Georgia	1				
APC		5.78	0.31	8.15	9.2
PC		5.69	0.42	8.26	7.3

^*a*^ The sample from the Imperial Valley, CA was included with the samples from Yuma, AZ, as described in the Results section.

^*b*^ There are 11 samples from Salinas, CA, area rather than 14 samples as indicated by Table 1 because (i) we were unable to accurately fit growth models for the 0422_B and 0522_A packaged samples as they were not tested until the end of shelf life (due to an incubator malfunction), and (ii) we were unable to confirm the location of cultivation for the 0922_B packaged sample.

We calculated Spearman correlation coefficients to quantify the strength of association of (i) the bacterial concentration at harvest and on D7 of shelf life, using data from harvest and packaged samples that were collected from the same order or lot, and (ii) the *N*_0_ and *N*_max_ of all packaged samples; we corrected the *P*-values for two comparisons, as specified above. The bacterial concentration of spinach at harvest and on D7 of shelf life was weakly correlated, given the following correlation coefficients: (i) 0.36, calculated using APC, and (ii) 0.14, calculated using PC; both coefficients were not significant (*P* > 0.05). However, the *N*_0_ and *N*_max_ of the packaged spinach were moderately correlated; these two parameters had the same correlation coefficient (0.69; *P* < 0.01) for estimates from primary growth models fit to APC and PC data. The moderate correlation between *N*_0_ and *N*_max_ suggests that the apparent difference in time to 7 log_10_ CFU/g between spinach from the Yuma and Salinas areas could be attributed to differences in N_0_. This is supported by the fact that the time to 7 log_10_ CFU/g was similar when assuming an *N*_0_ of 5 log_10_ CFU/g for all samples. Specifically, when assuming an *N*_0_ of 5 log_10_ CFU/g, the median (IQR) time to 7 log_10_ CFU/g was (i) 8.6 (7.6–12.5) days for the Yuma, AZ, area and 9.0 (7.3–11.9) days for the Salinas, CA, area, based on APC, and (ii) 7.7 (5.7–8.7) days for the Yuma, AZ, area and 7.3 (6.2–9.0) days for the Salinas, CA, area, based on PC.

### Higher preharvest temperatures are associated with increased bacterial levels on baby spinach

To assess the impact of weather on the difference in the bacterial concentration of baby spinach cultivated in the Yuma and Salinas areas, weather data were summarized for up to a week preceding harvest of the samples, clustered (to aid in stepwise forward selection, see Table 2 and Table S1), and included into the H model (i.e., the linear fixed effects model fit to the APC of the harvest samples). Following stepwise forward selection, the only weather variable retained in the H model was mean temperature 0–24 h prior to harvest; on average, a 1°C increase in mean temperature 0–24 h prior to harvest was associated with an increase in the APC of the harvest samples by 0.12 log_10_ CFU/g (*P* < 0.01). After accounting for weather (i.e., mean temperature 0–24 h prior to harvest) and duration from harvest until testing, harvest samples from the Salinas, CA, area, on average, showed a 0.20 log_10_ CFU/g higher APC than harvest samples from the Yuma, AZ, area (this difference was not significant, *P* = 0.35).

Weather variables were also added, as specified above, to the APC and PC models (i.e., linear mixed effects models fit to the APC and PC data of the packaged samples; see Table 2 and Table S2). Following stepwise forward selection of weather variables, minimum temperature 73–168 h prior to harvest was retained in both the APC and PC models; for this variable, a 1°C increase was associated (*P* < 0.05) with, on average, a 0.08 and 0.07 log_10_ CFU/g increase in APC and PC of packaged samples, respectively. Even after accounting for preharvest weather (i.e., minimum temperature 73–168 h prior to harvest), day of shelf life, and duration from harvest until arrival at the processing facility, the bacterial concentration of the packaged samples from the Salinas, CA, area was significantly higher than that of packaged samples from the Yuma, AZ, area, although the effect size was reduced to (i) 0.56 log_10_ CFU/g (*P* < 0.01), based on the APC of the packaged samples, and (ii) 0.47 log_10_ CFU/g (*P* = 0.05), based on the PC of the packaged samples.

Given that the *N*_max_ of the packaged spinach differed by growing region, and our prior analysis indicated that weather impacts the bacterial concentration of spinach, we used linear fixed effects models to assess the impact of weather on the *N*_max_ of the packaged spinach. Specifically, the dependent variable was the *N*_max_ of the packaged spinach, while the independent variable was the growing region; these models were fit separately to *N*_max_ estimates based on the APC and PC data of the packaged spinach. Weather variables were clustered (Table S2) and added as independent variables to these models using stepwise forward selection. The only weather variable retained in both models (i.e., fixed effects models of *N*_max_, based on APC and PC data) was minimum temperature 73–168 h prior to harvest, which was significantly (*P* < 0.05) associated with *N*_max_. On average, a 1°C increase in minimum temperature 73–168 h prior to harvest was associated with a 0.09 and 0.10 log_10_ CFU/g increase in *N*_max_ based on the APC and PC data, respectively. After accounting for the impact of weather (i.e., minimum temperature 73–168 h prior to harvest), we observed that the *N*_max_ of spinach from the Salinas, CA, area was significantly higher than that from the Yuma, AZ, area by 0.69 log_10_ CFU/g (*P* < 0.05), based on estimates from APC data. However, based on PC data, the *N*_max_ of spinach from the Salinas, CA, area was higher than that from the Yuma, AZ, area by 0.46 log_10_ CFU/g and was no longer significantly different (*P* > 0.05), after accounting for the impact of weather (i.e., minimum temperature 73–168 h prior to harvest).

### The genera of bacteria isolated from baby spinach cultivated in the Salinas, CA, area differed from those isolated from baby spinach cultivated in the Yuma, AZ, area

Of the 2,568 isolates that were selected from harvest and packaged samples in this study, we successfully sequenced the partial 16S rRNA gene of 2,397 isolates; we were unable to characterize the 16S rRNA gene of 171 isolates due to (i) logistical constraints (*n* = 103), which mainly consisted of an inability to culture isolates in brain-heart infusion (BHI; *n* = 92 of 103), and (ii) failure or poor quality of amplification or sequencing (*n* = 68). From the sequenced isolates (*n* = 2,397), we excluded the following isolates from statistical analysis: (i) 132 isolates that were collected from the microbiological tests of samples that were excluded from statistical analysis (i.e., 0722_B harvest sample, APC and PC of the 1222_A harvest sample, and 0922_B packaged sample), and (ii) 79 isolates that could not reliably be classified at the genus level; these 79 isolates represent the following order and families: (i) Enterobacterales (*n* = 43), (ii) Enterobacteriaceae (*n* = 21), (iii) Erwiniaceae (*n* = 8), (iv) Hafniaceae (*n* = 3), and (v) Caryophanaceae, Pectobacteriaceae, Micrococcaceae, and Yersiniaceae (*n* = 1 each). The remaining 2,186 characterized isolates represent 814 isolates from the Yuma, AZ, area, 1,205 from the Salinas, CA, area, 96 from FL, and 71 from GA. With regard to sample type, these isolates represent (i) 684 isolates from harvest samples, (ii) 840 from day 7 packaged samples (D7), (iii) 579 from day 22 packaged samples (D22), and (iv) 83 from day 28 packaged samples (D28).

**Fig 3:**
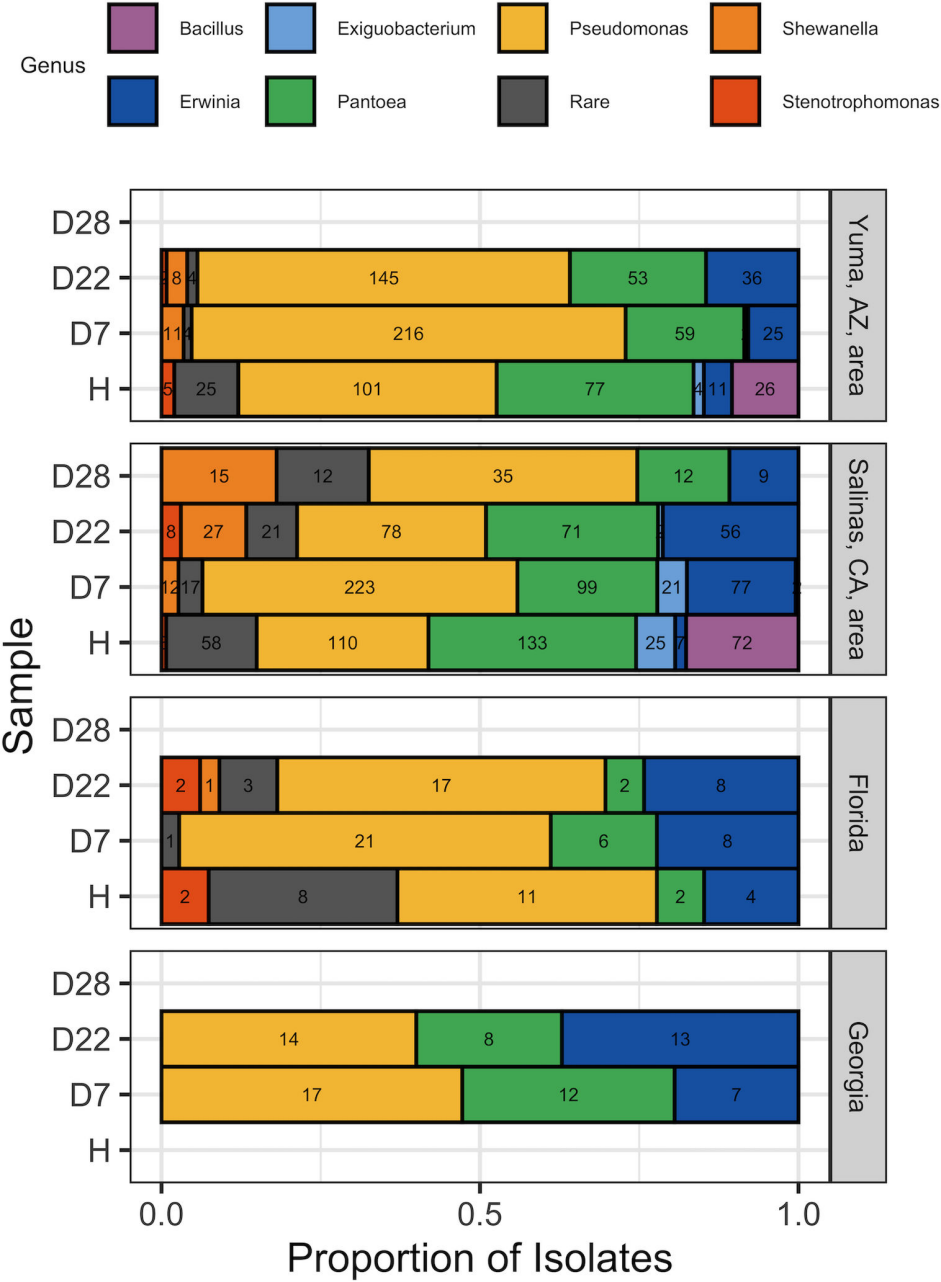
Proportion of the isolates classified as a given genus, by sample type and growing region; this plot was prepared with data from the isolates (*n* = 2,186 isolates) used for statistical analysis. The y-axis indicates the sample from which bacteria were isolated, as follows: (i) H represents harvest samples, while (ii) D7, D22, and D28 represent packaged baby spinach on days 7, 22, and 28 of shelf life, respectively (i.e., 7, 22, and 28 days postpackaging, with the day of packaging representing day 0). The x-axis represents the proportion of isolations classified as a given genus. Genus is indicated by color. The number of isolates classified as a given genus is specified within each bar. Any genera (or families or orders) that constituted <1% of isolates were re-classified as “rare”; this includes *Achromobacter, Acinetobacter, Aeromonas, Arthrobacter, Brevundimonas, Buttiauxella, Carnobacterium, Curtobacterium, Enterobacter, Enterococcus, Ewingella, Flavobacterium, Glutamicibacter, Hafnia, Kushneria, Lysinibacillus, Marinilactibacillus*, *Microbacterium, Paenibacillus, Peribacillus, Plantibacter, Pseudarthrobacter, Psychrobacter, Rahnella, Saccharibacillus, Sanguibacter, Serratia, Siccibacter, Sphingobacterium, Staphylococcus, Streptomyces,* and *Yersinia.*

Among the isolates used for analysis (*n* = 2,186), the most prevalent genera were *Pseudomonas* (*n* = 988, 45.2%), *Pantoea* (*n* = 534, 24.4%), and *Erwinia* (*n* = 261, 11.9%) (Fig. 3). These three genera are also the most prevalent, in the order listed above, among isolates from the Yuma, AZ, area and the Salinas, CA, area. Based on a Fisher’s exact test conducted using data from all 2,186 isolates, genus-level classification was associated with growing region (*P* < 0.01). For example, *Pseudomonas* spp. comprised 56.8% (*n* = 462) of the isolates from the Yuma, AZ, area but only 37.0% (*n* = 446) of the isolates from the Salinas, CA, area, and *Exiguobacterium* represented 0.7% (*n* = 6) of the isolates from the Yuma, AZ, area and 4.0% (*n* = 48) of the isolates from the Salinas, CA, area. Isolates classified as “rare” (i.e., isolates representing genera with a prevalence <1% among the 2,186 isolates) represented 4.1% and 9.0% of isolates from the Yuma and Salinas areas, respectively (33 and 108 isolates). *Pseudomonas* was also the most common genus among isolates from FL and GA (see Fig. 3 for details on isolates from FL and GA).

We also found that the genus-level classification of the isolates was associated with sample (i.e., harvest sample and packaged sample on D7, D22, and D28 of shelf life) (*P* < 0.01), based on a Fisher’s exact test conducted using data from all 2,186 isolates. Among isolates from harvest samples, the most prevalent genera were *Pseudomonas* (*n* = 222), *Pantoea* (*n* = 212), and *Bacillus* (*n* = 98). Similarly, among isolates collected from packaged samples on D7 and D22 of shelf life, the most prevalent genera were *Pseudomonas* (*n* = 477 from D7, and 254 from D22), *Pantoea* (*n* = 176 from D7, and 134 from D22), and *Erwinia* (*n* = 117 from D7, and 113 from D22). On D28 of shelf life, *Pseudomonas* was still the most prevalent genus (*n* = 35); however, the second and third most prevalent genera were *Shewanella* (*n* = 15) and *Pantoea* (*n* = 12); isolates classified as rare were also prevalent on D28 of shelf life (*n* = 12 isolates obtained on D28 of shelf life were classified as “rare”). Notably, isolates classified as rare appear to be relatively prevalent at harvest and at the end of shelf life (i.e., D22 or D28), as compared to D7 of shelf life. Specifically, the percentage of isolates classified as rare were (i) at harvest, 13.3% (*n* = 91) of the isolates; (ii) on D7 of shelf life, 2.6% (*n* = 22); (iii) on D22 of shelf life, 4.8% (*n* = 28); and (iv) on D28 of shelf life, 14.5% (*n* = 12).

**Fig 4:**
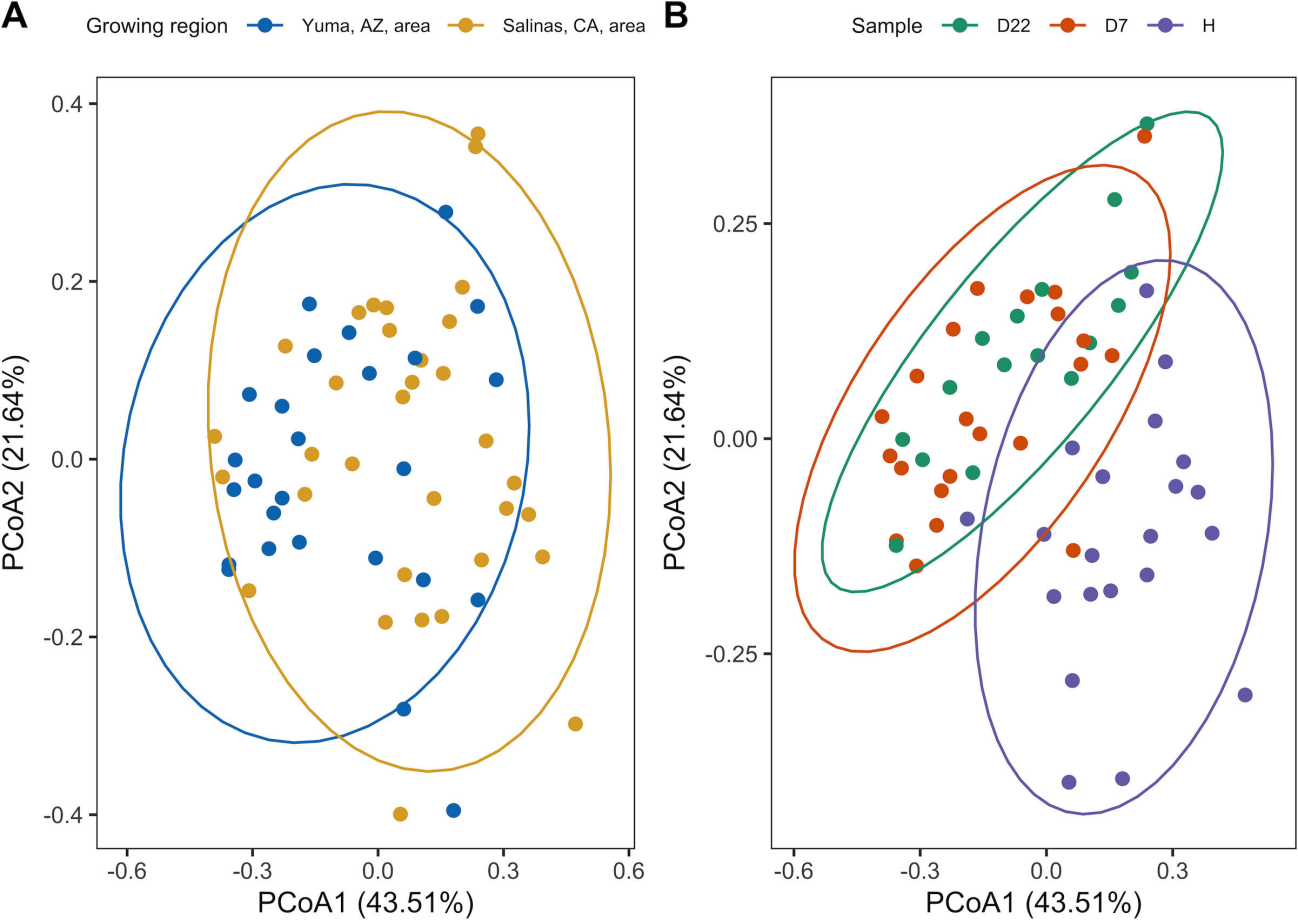
Principal coordinate analysis plot of the genus-level classification of the bacterial isolates used for the PERMANOVA and LefSe analysis. The y-axis and x-axis are the first and second principal coordinates, respectively; the percentage values represent the amount of variation in the data captured by a given principal coordinate. Color represents (i) growing region (A) and (ii) sample (B) (i.e., H is for spinach sampled after harvest, while D7 and D22 are day 7 and 22 of shelf-life testing for spinach sampled after packaging, respectively). Each data point represents the isolates gathered from a given sample; bacterial isolates collected on D28 of shelf life were excluded while preparing this plot to avoid using an imbalanced data set as bacteria were isolated on D28 of shelf life for only a few packaged samples from. Ellipses indicate 95% confidence intervals, based on the *t*-distribution.

As bacterial genera were associated with growing region and sample, a PERMANOVA was used to test whether bacterial genera were associated with the interaction of growing region (i.e., the Yuma and Salinas areas) and sample (i.e., harvest sample, and packaged sample on D7 and D22 of shelf life); we excluded isolates collected on D28 of shelf life from this analysis to avoid an imbalanced data set, as only a few packaged samples from the Salinas, CA, area were tested and subsequently subjected to bacterial isolation, on D28. Isolates from FL and GA were excluded from the PERMANOVA due to the low number of isolates for these two growing regions. The PERMANOVA found that bacterial genera were not significantly (*P* = 0.19) associated with the interaction of growing region and sample, although it confirmed that bacterial genera were significantly associated with growing region (*P* < 0.01) and sample (*P* < 0.01) separately (Table 4; Fig. 4). A linear discriminant analysis effect size (LEfSe) analysis, conducted using the data set used for the PERMANOVA, found that (i) *Pseudomonas* spp. were enriched in samples from the Yuma, AZ, area, while *Exiguobacterium* spp. were enriched in samples from the Salinas, CA, area, and (ii) *Erwinia* spp. and *Shewanella* spp. were enriched on D22 of shelf life.

**TABLE 4:** Summary of the PERMANOVA of bacterial isolates collected from AZ and CA

Coefficient	df	Sum of squares	*R* ^2^	*F*	Pr(>*F*)	Sig. level^*a*^
Sample	2	1.84	0.27	11.57	1.00 × 10^−3^	***
Growing region	1	0.49	0.07	6.14	2.00 × 10^−3^	**
Growing region: sample	2	0.23	0.03	1.42	0.19	NS
Residual	53	4.21	0.62	NA	NA	NA
Total	58	6.77	1.00	NA	NA	NA

^a^ The significance level represents the *P*-value: (i) *** for 0 ≤ *P* ≤ 0.001, (ii) ** for 0.001 < *P* ≤ 0.01, (iii) * for 0.01 < *P* ≤ 0.05, and (iv) . for 0.05 < *P* ≤ 0.1. NS represents *P*-values >0.10. NA represents “not applicable.” The PERMANOVA was run with seed set as “1.”

## DISCUSSION

In the U.S., AZ and CA are the leading producers of spinach. We assessed the bacterial quality of baby spinach, which was sampled between December 2021 and December 2022, from a supply chain that (i) mainly sources baby spinach from the Yuma, AZ, area and the Salinas, CA, area, and (ii) packages this baby spinach at a single processing facility. Our data suggest that the bacterial concentration of packaged baby spinach differs by growing region (i.e., Yuma and Salinas areas), supporting that growing region could be used as a proxy for additional and underlying factors that impact the bacterial concentration of spinach and illustrating the potential for season or region-specific strategies to manage the quality of this commodity. A broader finding was that differences in preharvest temperature partly contributed to the difference in the bacterial concentration of baby spinach by growing region; specifically, warmer preharvest temperatures were associated with an increase in the bacterial concentration on baby spinach after harvest and packaging. This suggests that produce cultivated in warmer growing regions (e.g., in the Salinas, CA, area during summer), as opposed to colder regions (e.g. in the Yuma, AZ, area during winter), may harbor larger bacterial populations. However, even after accounting for the impact of preharvest temperature, we observed that the bacterial concentration of packaged baby spinach from the Salinas, CA, area was still significantly higher than that of packaged baby spinach from the Yuma, AZ, area; this indicates that other factors, in addition to ambient temperature prior to harvest, may contribute to this region-based difference in bacterial concentration. Our data also suggest that the composition of the culturable spinach microbiota was associated with growing region (i.e., Yuma and Salinas areas); specifically, *Pseudomonas* spp. were enriched in spinach from the Yuma, AZ, area, while *Exiguobacterium* spp. were enriched in spinach from the Salinas, CA, area. Lastly, as this study only considered differences in bacterial concentration and populations on baby spinach, it is important that future studies understand how these differences in the bacterial quality of baby spinach, by growing region, may affect the development of sensory defects.

### Growing region impacts the bacterial concentration on baby spinach over shelf life

Our study found that baby spinach obtained from the Salinas, CA, area had a higher bacterial concentration over shelf life as compared to spinach obtained from the Yuma, AZ, area, even though bacterial numbers did not differ significantly for samples collected at harvest. By comparison, Rastogi et al. (17) conducted a study, on harvested lettuce (*n* = 106 samples), which found that the concentration of culturable bacteria was significantly higher, by 0.85 log_10_ CFU/g, for samples collected in summer (i.e., from Monterey County, CA) compared to those collected in winter (i.e., from the Imperial County, CA and Yuma, AZ). The difference in significance level between the study by Rastogi et al. (17) and our study could be due to the relatively low sample size in our study. Rastogi et al. (17) also reported that qPCR-based assessment of bacterial concentration did not find a significant difference between lettuce sampled in the summer (i.e., from Monterey County, CA) and that sampled in the winter (i.e., from the Imperial County, CA and Yuma, AZ). While culture-independent methods for bacterial quantification may facilitate more accurate enumeration by also accounting for viable-but-non-culturable bacteria, they can also overestimate bacterial numbers by accounting for dead bacteria (18). Our choice to quantify the bacterial concentration of baby spinach using only a culture-dependent method is justified, considering that (i) the food industry commonly uses culture-dependent microbiological methods (19, 20) and (ii) the concentration of culturable microbes has been shown to be associated with sensory defects in baby spinach (15).

Combined, our study as well as the study by Rastogi et al. (17) suggests that leafy greens cultivated in the Salinas, CA, area may have a higher concentration of culturable bacteria as compared to that cultivated in the Yuma, AZ, area. The difference in concentration of culturable bacteria, on leafy greens from the Yuma and Salinas areas, identified in our study and Rastogi et al. (17), was <1 log_10_ CFU/g. As this difference in bacterial concentration may seem low, we calculated the time to 7 log_10_ CFU/g for our samples of packaged spinach from the Yuma and Salinas areas, to contextualize this difference in bacterial concentration in more practical terms (e.g., difference in time to a certain bacterial threshold). Our analysis suggests that bacteria on packaged baby spinach from the Salinas, CA, area appear to reach 7 log_10_ CFU/g a few days earlier than that cultivated in the Yuma, AZ, area, although this difference was not significant. Notably, when we assumed a standard *N*_0_ of 5 log_10_ CFU/g, there was a negligible difference in time to 7 log_10_ CFU/g by growing region; this indicates that bacteria on packaged spinach from Salinas, CA, area appear to reach 7 log_10_ CFU/g faster than that from the Yuma, AZ, area due to a higher *N*_0_ (i.e., higher bacterial concentration on day 0 of shelf life) rather than a higher growth rate. While a lack of statistical significance indicates that the difference in time to 7 log_10_ CFU/g is not consistent and/or large between growing regions, it does not negate the possibility of a difference in the bacterial quality of packaged spinach from the Yuma and Salinas areas, particularly as the *N*_max_ for spinach differs significantly between samples from the Yuma and Salinas areas.

The difference in *N*_max_ between packaged baby spinach from the Yuma and Salinas areas suggests that bacteria on baby spinach from the Yuma, AZ, area may not grow to the same concentration as bacteria on baby spinach from the Salinas, CA, area regardless of storage duration, indicating a region-specific difference in bacterial dynamics on spinach that is more complex than a straightforward difference in bacterial concentration. For pure cultures of bacterial strains in defined media, transition to stationary phase can be triggered by cell-signaling behaviors such as quorum sensing, in response to environmental changes (e.g., exhausted nutrients) (21, 22), and has been shown to be associated with the expression of housekeeping genes (e.g., *rpoS* in gram-negative bacteria) (23). For mixed cultures of bacteria, other factors influence the transition to stationary phase, including the time at which the prevalent microorganism reaches stationary phase (24). Thus, bacterial communities on baby spinach from the Yuma and Salinas areas could differ in terms of factors that impact transition to stationary phase. Realistically, there are likely substantial differences in bacterial communities on baby spinach from the Yuma and Salinas areas, indicating the need for comprehensive genotypic and phenotypic characterization of the spinach microbiota by growing region.

In contrast to our findings, a study by Gu et al. (25) on the impact of chlorine washes on the spinach microbiota reported higher net bacterial growth on baby spinach from AZ, compared to that from CA, by 0.42 log_10_ CFU/g at 4°C; Gu et al. (25) specifically reported mean increases of (i) 2.25 log_10_ CFU/g on spinach from AZ and (ii) 1.83 log_10_ CFU/g on spinach from CA. For reference, the median net growth of our packaged samples, excluding those that could not be tested until the end of shelf life, was (i) 2.30 log_10_ CFU/g for the Yuma, AZ, area and 2.68 log_10_ CFU/g for the Salinas, CA, area, based on APC, and (ii) 2.48 log_10_ CFU/g for the Yuma, AZ, area and 2.96 log_10_ CFU/g for the Salinas, CA, area, based on PC. The fact that Gu et al. (25) only utilized spinach from a single lot for each AZ and CA could explain the differences in findings. Our analysis suggested that, on average, packaged baby spinach from the Salinas, CA, area had a higher bacterial concentration than packaged baby spinach from the Yuma, AZ, area. However, this observation would not always be true if we compared bacterial concentration of spinach from the Yuma and Salinas areas using data from one lot for each growing region, as some samples from the Yuma, AZ, area had higher bacterial concentrations than some samples from the Salinas, CA, area (see Fig. 2). Alternatively, although we collected multiple samples from the Yuma and Salinas areas, they were all sourced from ranches operated by a single company; thus, differences in the bacterial concentration on baby spinach from the Yuma and Salinas areas observed here could be supply chain (e.g., grower) specific. For example, a prior study (7) identified that the bacterial concentration on spinach can depend on cultivar type. We were unable to obtain information on the cultivar types assessed in our study. Hence, it is possible that the disagreement of our findings on the difference in bacterial concentration on spinach from AZ and CA, with that of Gu et al. (25), could be due to a difference in the cultivar types that were assessed. Ultimately, further work would be needed to assess whether, across ranches and supply chains, packaged spinach from the Salinas, CA, area has a higher bacterial concentration than packaged spinach from the Yuma, AZ, area. Based on our post hoc sample size calculation, we suggest a minimum of (i) 7 packaged samples from each the Salinas, CA, area and the Yuma, AZ, area (to replicate the findings of the APC model reported here), and (ii) 12 packaged samples from Salinas, CA, area and 8 packaged samples from the Yuma, AZ, area (to replicate the findings of the PC model reported here).

### Preharvest temperature partly explains the difference in the bacterial concentration of baby spinach by growing region

After accounting for the impact of preharvest temperature, we observed that the difference in the bacterial concentration of packaged baby spinach from the Yuma and Salinas areas was reduced but still significant (i.e., from 0.78 to 0.56 log_10_ CFU/g for the APC of packaged spinach, and from 0.67 to 0.47 log_10_ CFU/g for the PC of the packaged spinach). Comparatively, after accounting for preharvest temperature, we observed that the difference in the *N*_max_ of bacteria on packaged spinach, by growing region, was (i) reduced but still significant, for *N*_max_ values from models fit to APC data, and (ii) reduced and no longer significant, for *N*_max_ values from models fit to PC data. This suggests that some of the region-specific difference in the bacterial concentration of baby spinach could be due to weather differences between growing regions; given that growing regions are active during specific portions of a calendar year (e.g., the Yuma, AZ, area typically produces spinach in the winter), weather could be considered an “intrinsic” factor of growing region.

Specifically, our key finding was that high preharvest temperatures are associated with an increase in the (i) bacterial concentration on harvested and packaged baby spinach, and (ii) *N*_max_ of bacteria on packaged baby spinach. Previous studies have demonstrated that temperature is positively associated with the concentration of microbes on produce (9, 26). For example, a prior study (27) found that the average maximum temperature (up until 24°C), over 9 days preceding harvest, was positively associated with the concentration of *E. coli* on contaminated spinach, although increased maximum temperature was also negatively associated with the odds of spinach contamination with *E. coli*. Ultimately, even after accounting for preharvest temperature, there is still a region-dependent difference in the bacterial concentration of packaged baby spinach, indicating the need for additional research to identify other root cause(s) of this difference.

While one may surmise that higher bacterial levels on baby spinach at harvest, as caused by high preharvest temperatures, could lead to increased bacterial numbers throughout the supply chain, we did not find evidence to support this assumption, given the poor association between bacterial concentration at harvest and after packaging for spinach in our study reported here. Thus, there are likely additional cause(s) for the difference in the bacterial concentration of spinach from the Yuma and Salinas areas. Prior research has demonstrated that long-term weather patterns shape bacterial communities on plants (16), possibly to a greater extent than short-term weather patterns (28). It, thus, is also important to consider that long-term weather patterns may select for bacterial populations that, when introduced onto produce, differ in their capacity for cold growth (e.g., on refrigerated produce). This is plausible as the composition of the produce microbiota has been reported to be partly driven by horizontal transmission from the environment (e.g., soil) (29, 30). Prior studies have investigated “legacy effects” (long-term effects) of weather on the soil microbiome [e.g., elevated temperature (31) and drying-rewetting (32)]. For example, weather-related “legacy effects” may have selected for relatively cold-tolerant bacteria in the Salinas, CA, area that have a greater capacity for growth in cold environments (e.g., on refrigerated produce).

### Bacterial populations on baby spinach were associated with growing region, supporting the importance of region-specific characterization of bacterial spoilage for baby spinach

We also identified that bacterial communities on baby spinach differed significantly by growing region (i.e., the Yuma and Salinas areas); this is consistent with the findings of Rastogi et al. (17), which demonstrated that microbial populations on lettuce sampled in the summer growing season (i.e., from Monterey County, CA) and winter growing season (i.e., from Imperial County, CA and Yuma, AZ) were different. Specifically, Rastogi et al. (17) identified that (i) *Oxalobacteraceae* was more abundant in winter samples, while (ii) *Enterobacteriaceae* was more abundant in summer samples. In contrast, we identified that *Pseudomonas* spp. (family: *Pseudomonadaceae*) and *Exiguobacterium* spp. (family: *Bacillaceae*) were enriched on spinach cultivated in the Yuma and Salinas areas, respectively; the difference in enriched microorganisms, in our study compared to Rastogi et al. (17), could be attributed to the type of produce (i.e., spinach vs lettuce) as different vegetables have been reported to harbor different types of bacteria (33) or different proportions of similar bacteria (34). Overall, the combined findings, of our study and Rastogi et al. (17), support that bacterial populations on produce can be associated with growing region.

We identified *Pseudomonas* and *Pantoea* to be the most abundant genera on baby spinach. This observation is partly supported by prior studies on the spinach microbiota, which have reported varying levels of these two genera. At harvest, a prior study (35) identified *Pantoea* as one of the most abundant genera on spinach (i.e., at least 13.34% of the amplicons), while another study (36) reported relatively high levels of *Pseudomonas* (6.57% and 31.7% of amplicons, in autumn and spring, respectively). Thus, we observed *Pseudomonas* spp. and *Pantoea* spp. at higher levels on harvested spinach (i.e., both genera represented around 30% of the isolates from harvest samples in this study) than commonly reported in the literature. Conversely, our finding that *Pseudomonas* is the most abundant genus on packaged spinach is supported by prior studies (35, 36), which also reported that *Pseudomonas* is the most abundant bacterial genus on spinach during refrigerated storage. To our knowledge, only one study (35) reported that *Pantoea* was the second most abundant genus on spinach during refrigerated storage. As our study used culture-dependent characterization of the spinach microbiota, while the aforementioned studies (35, 36) utilized culture-independent characterization, the difference between our findings and prior studies suggests a value for future culture-independent characterization of bacterial populations on spinach. Such studies will provide insight into whether the variability of our findings, compared to that of prior studies, is due to (i) differences in our approach to characterizing the spinach microbiota or other factors, such as (ii) sample variability, given that the study by Rastogi et al. (17) found a large difference in the prevalence of *Pantoea* spp. on lettuce after controlling for the presence of *Xanthomonas* spp.

Importantly, our findings also suggest that additional work is necessary to identify key spoilage microorganisms or processes in produce. While we identified that the bacterial concentration of packaged spinach from the Yuma and Salinas areas may differ, at most, by 0.78 log_10_ CFU/g (i.e., difference in APC without accounting for the effect of weather, see Table 2), solely focusing on differences in bacterial concentration may not provide a comprehensive insight into the postharvest quality of baby spinach. Specifically, a surprising finding was that *Pseudomonas* spp., which are regarded as important spoilage microorganisms in produce (37, 38), were enriched in samples from the Yuma, AZ, area, although samples from that area had a lower bacterial concentration over shelf life, compared to samples from the Salinas, CA, area. This contrast of the higher bacterial concentration but lower prevalence of *Pseudomonas* spp. for spinach from the Salinas, CA, area demonstrates the need to identify whether the (i) overall bacterial concentration or (ii) prevalence of key microorganisms (i.e., *Pseudomonas* spp.) may be a more accurate indicator of poor postharvest quality; furthermore, it highlights the need to understand how these differences (e.g., in bacterial concentration) may impact the development of sensory defects. Thus, it is important for future studies to not only consider the mathematical significance (i.e., difference in bacterial concentration) when comparing the postharvest bacterial quality of produce but also the biological relevance of such findings (e.g., prevalence and impact of key microorganisms on postharvest decay). More broadly, this finding (on the enrichment of *Pseudomonas* spp. on spinach from the Yuma, AZ, area) also highlights the need to assess other genera, in addition to *Pseudomonas*, as microbiological markers of high bacterial concentration and possibly spoilage, for baby spinach; two potential microbiological markers, based on our study, were *Erwinia* and *Shewanella*, as these two genera were enriched on D22 of shelf life.

In addition to our study, which found that *Shewanella* represented 3.2% (*n* = 8 of 248) and 10.3% (*n* = 27 of 263) of the isolates obtained from the Yuma and Salinas areas on D22 of shelf life, respectively, a previous study (36) reported *Shewanella* spp. at low levels (<3.07% of amplicons) on spinach that was harvested in autumn or spring and tested at the end of shelf life. Although many studies have focused on the spoilage potential of *Shewanella* spp. in seafood (39, 40), our data suggest that this genus may also play a role in the quality of fresh produce as *Shewanella* spp. were prevalent later in shelf life and were numerically more abundant on spinach from the Salinas, CA, area, which also had higher bacterial levels than spinach from the Yuma, AZ, area. However, additional research is needed to identify whether the increased prevalence of *Shewanella* spp. on spinach from the Salinas, CA, area is reproducible and if so is due to more rapid bacterial growth or higher rates of introduction from the growing environment. As *Shewanella* are aquatic bacteria (41), which were previously shown to be relatively prevalent in sections of the Pacific Ocean (42), it is possible that the increased prevalence of *Shewanella* spp. on spinach from Salinas, CA, area could be due to increased introduction of these bacteria into the Salinas, CA, area, which is close to the Pacific Ocean.

In addition to *Shewanella* spp., we also identified that *Erwinia* spp. were enriched on D22 of shelf life; thus, *Erwinia* spp. could also be a potential marker of or contributor to high bacterial levels (and possibly microbial spoilage) of leafy greens, as supported by a prior study (36) that reported *Erwinia* spp. at low levels (<3.64% of amplicons) on rocket that was harvested in spring and tested at the end of shelf life. Prior research has demonstrated that *Erwinia* spp. produce pectinolytic enzymes (43) and are associated with soft rot in produce (44, 45), which could explain why this genus is enriched on D22 of shelf life, as production of pectinolytic enzymes would likely allow for an increased ability to access nutrients within plant cells. Further research is necessary to understand the extent to which cell wall-degrading enzymes (e.g., pectinolytic enzymes) may (i) enhance bacterial growth on produce and (ii) contribute to postharvest quality defects. Combined with our data, this type of future research may provide further insights into specific bacteria that could be targeted for control in order to reduce spoilage, for example, by using biocontrol strategies at the pre- or postharvest stage. Importantly, biocontrol agents (including some bacteria commonly associated with spoilage, e.g., *Pseudomonas*) to manage plant diseases and promote plant growth in the preharvest environment have been identified (46) and are commercially available. This illustrates the potential importance of assessing whether organisms used in biocontrol may contribute to or cause postharvest spoilage.

### Conclusions

The findings of this study demonstrate a region-specific difference in the bacterial quality of baby spinach and thus could possibly inform region-specific quality management practices; for example, the data from this study could be used to inform (i) shelf-life dating, based on growing region, and/or (ii) prioritizing inventory management based on preharvest temperatures between and within growing regions rather than using a first-in-first-out system. As warm preharvest temperatures only partly explained this region-specific difference in bacterial concentration, further research is needed to identify additional root cause(s) of this difference. It is likely that the observed region-specific difference in bacterial concentration on baby spinach is partly due to differences in bacterial community composition rather than solely due to a difference in bacterial loads, given evidence of region-specific differences in bacterial populations on spinach, including the capacity for cold growth of members of these populations. Thus, these findings highlight the need for further characterization of changes in bacterial communities on baby spinach over shelf life by growing region and diverse patterns of underlying environmental conditions, including assessing their impact on the development of spoilage defects; such data are important to better assess the need for region-specific postharvest quality management strategies.

## MATERIALS AND METHODS

### Sample collection

From December 2021 through December 2022, we sampled baby spinach from a supply chain, which comprises a single processing plant that mainly sources baby spinach from a grower based in the Yuma, AZ, area (including Yuma, AZ and Imperial Valley, CA) and the Salinas, CA, area. Some samples were collected from a grower based in FL and GA, which also delivers product to the same processing plant. In this supply chain, spinach was washed with chlorine, at the processing facility, and subsequently packaged (i.e., as whole, unprocessed produce) for distribution. Approximately two samplings were conducted each month from this supply chain; for each sampling, spinach was collected after harvest (harvest samples) and packaging (packaged samples), typically from the same order (i.e., lot). Harvest samples consisted of three 500 g portions of baby spinach that were collected using gloved hands by the growers and transferred to separate sterile Whirlpak bags (Nasco, Fort Atkison, WI). Packaged samples were collected by the processor and consisted of either three 141 g (i.e., 5 oz.) bags, three 453 g (i.e., 1 lb.) bags, or one 1,134 g (i.e., 2.5 lb.) bag of spinach. All samples were shipped overnight, in coolers filled with ice packs, to Ithaca, NY. Samples were covered in bubble wrap prior to shipping, to avoid bruising and direct contact with ice packs. Temperature loggers (DeltaTRAK, Modesto, CA & Omega, Norwalk, CA) were used to monitor the cooler temperature during shipment to Ithaca, NY; the average temperature during the apparent shipment time period was below 6°C for all samples.

We administered surveys, electronically (via email) or through printed forms, to the growers and the processor to obtain metadata for the samples. For each harvest sample, we gathered data, including: (i) location of the ranch, from where the spinach was harvested, (ii) date of the last irrigation, and (iii) date of harvest. For each packaged sample, we collected data, including: (i) date of arrival at the processing facility, (ii) temperature on arrival at the processing facility, (iii) the type of antimicrobial wash used prior to packaging, and (iv) date of packaging. For packaged samples that were collected from orders (i.e., lots), from which we did not collect harvest samples, we administered a survey to the grower to collect field-level data (specifically, the location of the ranch from where the spinach was harvested and the date of harvest).

### Microbiological testing

All samples were tested, in triplicate, on arrival in Ithaca, NY; for packaged samples, testing on arrival represented day “initial” of shelf life. Subsequently, 25 g portions of each packaged sample were transferred to sterile Whirlpak bags and stored at 4°C for shelf-life testing, in triplicate, on day 7 of shelf life (i.e., 7 days after the day of packaging, D7) and subsequently every 5 or 7 days until day 22 (D22) or day 28 (D28) of shelf life, respectively. For testing, 25 g of spinach was stomached (Stomacher 400 Circulator, Seward, Bohemia, NY), at 230 beats per minute for 2 min, in a sterile Whirlpak bag with approximately 200 mL of Butterfield’s phosphate buffer (BPB). Two samples were stomached with 225 mL of BPB; however, the buffer volume was accounted for when calculating the bacterial concentration of the samples. The resulting sample homogenates were serially diluted in BPB and subsequently plated, in duplicates, onto the following Petrifilms (3M, Saint Paul, Minnesota): (i) aerobic count Petrifilm (twice), with one pair incubated at 35°C for 48 h (APC) and the other pair incubated at 7°C for 10 days (PC), and (ii) coliform count Petrifilm, incubated at 35°C for 48 h, before enumerating all colonies regardless of gas production (total GN) (47).

Bacteria were isolated from the Petrifilms of (i) harvest samples; (ii) packaged samples, tested on D7; and (iii) packaged samples, tested at the end of shelf life (i.e., D22 or D28); two colonies were selected from each Petrifilm, resulting in 36 colonies per sample [2 colonies per Petrifilm × 2 Petrifilm replicates per test × 3 tests (APC, PC, GN) per replicate × 3 replicates per sample]. Colony morphology did not influence the selection process, as colonies typically have a uniform morphology on the Petrifilms used in this study; thus, colonies were selected conveniently without considering colony phenotype. To obtain isolates, the selected colonies were substreaked onto brain-heart infusion agar plates (Becton, Dickson and Company, Franklin Lakes, NJ), which were incubated at 32°C for 24 h. If a mixed culture was present on a BHI plate, pure cultures were obtained by substreaking each observed morphology onto new BHI plates. For each isolate, a colony from the substreaked BHI plate was used to inoculate 5 mL of BHI broth, which was incubated at 32°C until cloudy (typically, 24–72 h); an aliquot of the BHI broth culture was combined with sterile glycerol (15% vol glycerol/vol culture), and stored at −80°C. Isolate data are available on Food Microbe Tracker (48) (www.foodmicrobetracker.com).

Isolates were characterized by Sanger sequencing of the 16S rRNA gene. Isolates were substreaked from frozen stocks onto BHI plates, which were incubated at 32°C for 24–72 h. Lysates for PCR were prepared by suspending a partial colony of each isolate in separate 100 µL aliquots of sterile, ultrapure water and heating the resulting suspension to 95°C for 5 min. As previously described (49), a partial fragment of the 16S rRNA gene was amplified using primers PEU-7 and DG-74; post-PCR cleanup was conducted with exonuclease I and shrimp alkaline phosphatase. The PCR products were subsequently submitted to Biotechnology Resource Center (Cornell University, Ithaca, NY) for Sanger sequencing, which consisted of (i) chain termination PCR with the BigDye v.3.1 Kit (ThermoFisher, Waltham, MA), (ii) subsequent cleanup, and (iii) sequencing with the 3730xl DNA Analyzer (Applied Biosystems, ThermoFisher, Waltham, MA).

### Data analysis

All analyses were conducted in R v.4.2.2 (50). Figures were prepared with the ggplot2 package (51) in R. For each sample, the date and time of harvest were used to obtain hourly weather data for the week preceding harvest from Visual Crossing (https://www.visualcrossing.com/). We were unable to obtain the time of harvest for a few samples (*n* = 8 samples); for these eight samples, we used the median time of harvest (i.e., 3:00 am) of the remaining samples in our data set when downloading weather data for the week preceding harvest. Weather metrics were calculated, for harvest and packaged samples, during the following time intervals preceding harvest: (i) 0–24 h, (ii) 25–48 h, (iii) 49–72 h, and (iv) 73–168 h (i.e., 4–7 days preceding harvest); weather metrics included (i) mean, minimum, and maximum temperature (in degree Celsius); (ii) mean dew (in degree Celsius); (iii) mean windspeed (in kilometer per hour); and (iv) mean solar radiation (in watts per square meter). Total precipitation (in millimeter) was summarized for 0–72 h and 73–168 h preceding harvest. The geometric mean bacterial concentration of the samples was calculated by averaging the log_10_-transformed bacterial concentration of the sample replicates; the geometric mean bacterial concentration data were used for subsequent analysis. The bacterial concentration and isolate data from the following samples were excluded from statistical analysis: (i) a harvest sample (0722_B, see Table 1), which was delayed in shipment for 4 days, (ii) APC and GN tests of a harvest sample (1222_A, see Table 1), which were incubated at the wrong temperature, and (iii) a packaged sample (0922_B, see Table 1), for which we were unable to collect metadata (e.g., growing region). We also excluded data from samples cultivated in FL and GA from statistical analysis, as we only had (i) one harvest and packaged sample from FL and (ii) one packaged sample from GA (Table 1).

We calculated the median and interquartile range of bacterial concentration using the “quantile” function in R (50); the “quantile” function was specified with “type = 7,” which results in the use of linear interpolation to calculate sample quantiles. To assess whether the bacterial concentration of baby spinach was associated with growing region, we fit a linear mixed effects model separately to data from harvest samples (H model) and packaged samples (P model) using the “lmer” function from the LmerTest package (52); random effects were included to account for repeated measures, including: (i) the use of multiple microbiological tests (APC, PC, and GN) to measure bacterial concentration and (ii) repeated testing of packaged samples over shelf life. Fixed effects of the interaction of test (APC, PC, or GN) and growing region were included in the H and P models, to assess if the outcome of the model (specifically, the effect of growing region on bacterial concentration) differs among APC, PC, and GN data sets; if this interaction was significant, models were fit separately to data from the individual tests (i.e., APC and PC models, see Results). If this interaction was not significant, models were only fit to the APC of the samples as the outcome of our hypothesis testing (i.e., whether bacterial concentration on spinach differed by growing region) was not dependent on the measure of bacterial concentration (e.g., APC or PC); APC was selected for further analysis because tests for aerobic, mesophilic bacteria are commonly used to assess the quality of food products in industry (53). Additionally, if we did not need to account for repeated measures, such as the use of multiple microbiological tests (i.e., for the H model, see Results), the “lm” function in R (50) was used to fit a linear fixed effects model.

Both H and P models used bacterial concentration of the samples (in log_10_ CFU/g) as the dependent variable and growing region as an independent variable (i.e., as a fixed effect). Additionally, the H and P models also included the following independent variables (i.e., as fixed effects): (i) H model: (*a*) time from harvest until testing and (*b*) days since last irrigation, and (ii) P model: (*a*) time from harvest until arrival at the processing facility, (*b*) time from arrival at the processing facility until packaging, (*c*) day of shelf life, and (*d*) the interaction of growing region and variables (*a*) and (*b*) of the P model. Day of shelf life was included in the P model as a second-degree polynomial, using the “poly” function in R (50), to account for the non-linear increase in bacterial concentration over shelf life. The “step” function from the lmerTest package (52) was used for backward variable selection, by removing variables that did not significantly improve model fit (i.e., had a *P*-value >0.10); growing region was not removed during backward variable selection, as it was the focus of our hypothesis (i.e., *H*_0_: bacterial concentration does not differ by growing region). Additionally, the linear mixed effects models were fit with maximum likelihood during model selection, and the final model (e.g., after backward variable selection) was fit with restricted maximum likelihood. To assess whether bacterial concentration at harvest was associated with bacterial concentration over shelf life, we fit the P model to a subset of data (i.e., data from orders or lots, from which we collected both harvest and packaged samples); for this “reduced P model,” we included bacterial concentration at harvest as an independent variable, in addition to the other independent variables as described above for the P model.

We used the following criteria to select weather variables to include in the H model and P model: (i) weather variables were added, one at a time, to the H or P model, followed by measuring the Akaike Information Criteria (AIC) of the model using the “aic” function from the stats package (50), (ii) weather variables were clustered using the “mlcc.bic” function from the varclust package (54) while specifying “numb.clusters” as 1–7 and “numb.runs” as 20, and (iii) for each resulting cluster, two weather variables, which resulted in the lowest model AIC, were selected for stepwise forward selection; variables that were not assigned to any cluster were also selected for stepwise forward selection. If selected weather variables had a Spearman correlation coefficient greater than 0.70, as assessed using “cor.test” function from the stats package (50), an alternative weather variable was selected for inclusion in the model (i.e., to replace whichever of the two selected weather variables resulted in a lower improvement of model fit as estimated by AIC). Subsequently, stepwise forward selection was conducted, starting with weather variables that led to the lowest model AIC; variables were retained in the model if they improved model fit (i.e., had a *P*-value <0.10) as measured using the “step” function. The mixed effects models were fit with maximum likelihood during forward selection of weather variables, and the final model was fit with restricted maximum likelihood. The “cor.test” function from the stats package (50) was used to calculate the Spearman correlation coefficient of bacterial concentration at harvest and on D7 of shelf life (i.e., the day of shelf life that all packaged samples were tested) using data from harvest and packaged samples that were collected from the same order (i.e., lot); the Holm method was used to correct for multiple comparisons (see Results), using the “p.adjust” function from the stats package (50).

The following primary growth models, from the nlsMicrobio package (55), were fit to the shelf-life data of the packaged samples using the “nlsLM” function from the minpack.lm package (56): (i) Baranyi without lag phase (57) and (ii) Buchanan without lag phase (58); bacterial concentration (in log_10_ CFU/g) was the dependent variable, and day of shelf life was the independent variable. For each packaged sample, the primary growth model with the lowest AIC was selected to obtain growth parameters, including: (i) initial bacterial concentration (*N*_0_), which is the bacterial concentration on the day of packaging (i.e., day 0 of shelf life), (ii) bacterial growth rate (*µ*_max_), and (iii) bacterial concentration at stationary phase (*N*_max_). The “wilcox.test” function, from the stats package (50), was used to conduct Mann-Whitney *U* tests to compare growth parameters by growing region with correction for multiple comparisons as described above; for growth parameters that varied significantly by growing region, we assessed the impact of weather using linear fixed effect models that were fit with the “lm” function as described above. We also calculated the Spearman correlation coefficient of the *N*_0_ and *N*_max_ of the packaged samples to understand whether bacterial concentration early in shelf life is associated with bacterial concentration later in shelf life, using the “cor.test” function as described above; growth parameters were used for this calculation rather than the bacterial concentration of the packaged samples, as they were a consistent metric for comparison, while bacterial concentration data varied by sample (e.g., day initial, which was the first day of shelf-life testing, varied by sample). The growth parameters of the packaged samples were also used to calculate time to 7 log_10_ CFU/g, with the “time_to_size” function from the biogrowth package (59). Time to 7 log_10_ CFU/g was compared by growing region, using a Mann-Whitney *U* test with correction for multiple comparisons as described above.

The 16S rRNA gene sequences of the isolates were assembled and proofread in Sequencher v.5.4.6 (Gene Codes, Ann Arbor, MI). A BLAST search of the sequences was conducted against a local copy (downloaded on 16 November 2022) of the 16S rRNA gene database from the Ribosomal Database Project (60), to assign genus-level taxonomic classifications to isolates based on percent identity match from the BLAST results; the BLAST search was conducted with a maximum number of target sequences (i.e., number of results per isolate) specified as 6. The output from the BLAST search was filtered to remove results for which the alignment length (of the query and subject sequences) was <99% of the query length (i.e., the sequence length of a given isolate). Isolates were subsequently classified at the genus level by assigning the genus of an isolate’s top BLAST match based on percent identity (i.e., the BLAST match that had the highest percent identity). An isolate was classified at a higher taxonomic level than genus if, relative to the isolate’s top BLAST match, the isolate had another match that was (i) from a different genus than the top match and (ii) within 0.50 percentage points of the percent identity of the top match. Isolates that could not be classified at the genus level were left out of statistical analysis. Additionally, genera that comprised less than 1% of the isolates were classified as “rare,” prior to statistical analysis. Fisher’s exact tests were conducted, using the “fisher.test” function from the stats package (50) with *P*-values computed by Monte Carlo simulation, to determine whether bacterial genera were associated with: (i) growing region or (ii) sample (i.e., harvest sample, or packaged sample on D7, D22, or D28). A PERMANOVA was conducted using the “adonis2” function from the vegan package (61) to test whether bacterial genera were associated with the interaction of growing region and sample; for the PERMANOVA, isolate data were used to construct a Bray-Curtis dissimilarity matrix (BC matrix) using the “vegdist” function from the vegan package. Isolates collected on D28 of shelf life were excluded from the PERMANOVA to avoid an imbalanced data set as only a few samples from the Salinas, CA, area were tested and subjected to bacterial isolation, on D28 of shelf life (see Table 1). The PERMANOVA was run with 999 permutations, specifying the BC matrix as the dependent variable, and growing region and sample as the independent variables. Linear discriminant analysis effect size was implemented, using the “run_lefse” function from the microbiomeMarker package (62) and the data set used for the PERMANOVA, to identify genera that were enriched by growing region or sample separately; for the LEfSe, the LDA cutoff was specified as “4,” and multigrp_strat was specified as “TRUE”.

### Post hoc sample size calculation

We conducted a post hoc sample size calculation to identify, based on our data, the minimum number of packaged samples that would be necessary to replicate this study with an alpha of 0.05 and power of 80%. To obtain these values, we conducted a simulation-based sample size calculation; for this calculation, we used (i) a minimum of 4 packaged samples from the Yuma and Salinas areas, up to (ii) a maximum of 8 packaged samples from the Yuma, AZ, area and 12 packaged samples from the Salinas, CA, area. This resulted in 45 possible combinations (e.g., 5 samples from the Yuma, AZ, area and 7 from the Salinas, CA, area) of sample numbers from the Yuma and Salinas areas. For each combination of sample numbers, we simulated a random draw (without replacement) of packaged samples from our data set and used these randomly selected samples to fit the mixed effects model specified above (i.e., the P model, without weather). The random draw and model fitting were simulated 100 times for each combination of sample numbers from the Yuma and Salinas areas; the seed was set as “1” for the simulation. Power was calculated as the percentage of 100 simulations in which the null hypothesis (*H*_0_: bacterial concentration does not differ by growing region) was rejected with an alpha of 0.05.

## Data Availability

The data and R code for this study are available on Github: https://github.com/FSL-MQIP/2022_spinach_sampling_counts_isolates.

## SUPPLEMENTAL MATERIAL

Supplemental material aem.00553-24-s0001.docx Figures S1 to S3; Tables S1 and S2.

ASM does not own the copyrights to Supplemental Material that may be linked to, or accessed through, an article. The authors have granted ASM a non-exclusive, world-wide license to publish the Supplemental Material files. Please contact the corresponding author directly for reuse.
