# Supplementary material for: A longitudinal study on the bacterial quality of baby spinach cultivated in Arizona and California
Source: Appl Environ Microbiol. 2024 Jul 12;90(8):e00553-24. doi: 10.1128/aem.00553-24 (PMC11337821; doi:10.1128/aem.00553-24)
Supplement: Supplemental material — Figures S1 to S3; Tables S1 and S2. [file aem.00553-24-s0001.docx]

**Supplemental Materials**

**Supplemental Figure 1:** Bacterial concentration of packaged baby spinach (in log_10_ CFU/g; y-axis) on day 7 of shelf life, by test. For plot A (scatterplot), the x-axis is the time of sampling (YYYY-MM) and y-axis is the bacterial concentration of a sample. For plot B (boxplot), the x-axis that represents the growing region and the y-axis is bacterial concentration; this plot only represents samples from the Yuma, AZ, area and the Salinas, CA, area. Color represents growing region. The data are depicted separately by test, as indicated by the following labels: (i) aerobic plate count (APC), (ii) total Gram-negative count (GN), and (iii) psychrotolerant count (PC). The 0922_B packaged sample was excluded from this plot, as we were unable to determine its location of cultivation.


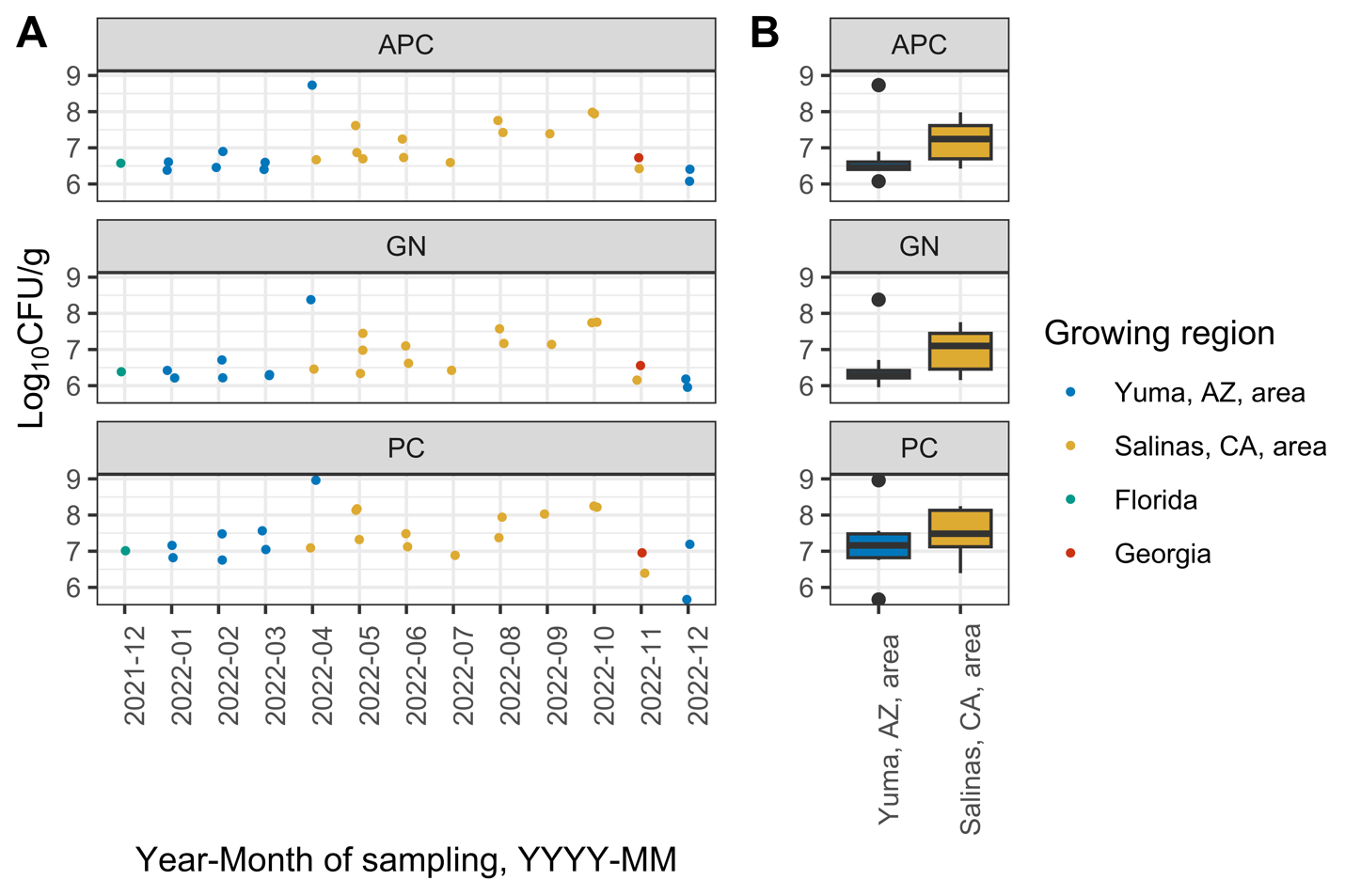


**Supplemental Figure 2:** Plot of primary growth models that were fit to the APC data of packaged spinach samples that were incubated at 4˚C. The y-axis is bacterial concentration, and the x-axis is the day of shelf life (i.e., the day post-packaging, with the day of packaging representing day 0 of shelf life). Points represent observed data, and point shape represents technical replicate of a given packaged sample. The lines represent the prediction of the primary growth model that was fit to a given packaged sample. Plot titles represent the sampling ID of the packaged samples. We were unable to determine the growing region for the 0922_B packaged sample.

**Supplemental Figure 3:** Plot of primary growth models that were fit to the PC data of packaged spinach samples that were incubated at 4˚C. The y-axis is bacterial concentration, and the x-axis is the day of shelf life (i.e., the day post-packaging, with the day of packaging representing day 0 of shelf life). Points represent observed data, and point shape represents technical replicate of a given packaged sample. The lines represent the prediction of the primary growth model that was fit to a given packaged sample. Plot titles represent the sampling ID of the packaged samples. We were unable to determine the growing region for the 0922_B packaged sample.

**Supplemental Table 1:** Weather variables that were summarized for the harvest samples, grouped by cluster. Variables marked by an asterisk were selected for addition to the H model.

| Cluster 1 | Cluster 2 | Did not cluster with other variables |
| --- | --- | --- |
| Mean windspeed (km/h), 49 to 72 h prior to harvest (pth) ^*^ | Maximum temperature (˚C), 0 to 24 h pth | Sum of precipitation (mm), 0 – 72 h pth^*^ |
| Sum of precipitation (mm), 73 – 268 h pth^*^ | Maximum temperature (˚C), 25 to 48 h pth |  |
|  | Maximum temperature (˚C), 49 to 72 h pth |  |
|  | Maximum temperature (˚C), 73 to 168 h pth |  |
|  | Mean dew (˚C), 0 to 24 h pth |  |
|  | Mean dew (˚C), 24 to 48 h pth |  |
|  | Mean dew (˚C), 49 to 72 h pth |  |
|  | Mean dew (˚C), 73 to 148 h pth |  |
|  | Mean solar radiation (W/m2), 0 to 24 h pth |  |
|  | Mean solar radiation (W/m2), 25 to 48 h pth |  |
|  | Mean solar radiation (W/m2), 49 to 72 h pth |  |
|  | Mean solar radiation (W/m2), 73 to 148 h pth |  |
|  | Mean temperature (˚C), 0 to 24 h pth^*^ |  |
|  | Mean temperature (˚C), 24 to 48 h pth |  |
|  | Mean temperature (˚C), 49 to 72 h pth |  |
|  | Mean temperature (˚C), 73 to 168 h pth |  |
|  | Mean windspeed (km/h), 0 to 24 h pth |  |
|  | Mean windspeed (km/h), 25 to 48 h pth |  |
|  | Mean windspeed (km/h), 73 to 168 h pth |  |
|  | Minimum temperature (˚C), 0 to 24 h pth |  |
|  | Minimum temperature (˚C), 25 – 48 h pth |  |
|  | Minimum temperature (˚C), 49 to 72 h pth^*^ |  |
|  | Minimum temperature (˚C), 73 to 168 h pth |  |

**Supplemental Table 2:** Weather variables that were summarized for the packaged samples, grouped by cluster. Variables marked by an asterisk, plus, pound sign and apostrophe were selected for addition to, respectively, the models fit to the (i) APC, (ii) PC, (iii) N_max_ of APC of the packaged samples, and (iv) N_max_ of the PC of the packaged samples. NOTE: The clusters of weather variables for the packaged samples differ from those of the harvest sample, as some packaged samples had different harvest dates and thus, different preharvest weather that some harvest samples; only 17 harvest and packaged samples were collected from the same order or lot and share the same preharvest weather data.

| Cluster 1 | Cluster 2 |
| --- | --- |
| Sum of precipitation (mm), 0 – 72 h, prior to harvest (pth)*^+#^’ | Maximum temperature (˚C), 0 to 24 h pth |
| Sum of precipitation (mm), 73 – 168 h pth *^+#^’ | Maximum temperature (˚C), 24 to 48 h pth |
|  | Maximum temperature (˚C), 49 to 72 h pth |
|  | Maximum temperature (˚C), 73 to 168 h pth |
|  | Mean dew (˚C), 0 to 24 h pth |
|  | Mean dew (˚C), 24 to 48 h pth |
|  | Mean dew (˚C), 49 to 72 h pth |
|  | Mean dew (˚C), 73 to 168 h pth |
|  | Mean solar radiation (W/m^2^), 0 to 24 h pth |
|  | Mean solar radiation (W/m^2^), 25 to 48 h pth |
|  | Mean solar radiation (W/m^2^), 49 to 72 h pth^#^’ |
|  | Mean solar radiation (W/m^2^), 73 to 168 h pth |
|  | Mean temperature (˚C), 0 to 24 h pth |
|  | Mean temperature (˚C), 24 to 48 h pth |
|  | Mean temperature (˚C), 49 to 72 h pth |
|  | Mean temperature (˚C), 73 to 168 h pth |
|  | Mean windspeed (km/h), 0 to 24 h pth |
|  | Mean windspeed (km/h), 25 to 48 h pth |
|  | Mean windspeed (km/h), 49 to 72 h pth *^+^ |
|  | Mean windspeed (km/h), 73 to 168 h pth |
|  | Minimum temperature (˚C), 0 to 24 h pth |
|  | Minimum temperature (˚C), 25 – 48 h pth |
|  | Minimum temperature (˚C), 49 to 72 h pth |
|  | Minimum temperature (˚C), 73 to 168 h pth *^+#^’ |
